# Challenges facing community health workers in promoting maternal and neonatal health in Bagamoyo and Mkuranga districts, Tanzania

**DOI:** 10.4314/gmj.v55i2.4

**Published:** 2021-06

**Authors:** Kabula Jumanne, Liliane Pasape, Irene R Moshi, Mwifadhi Mrisho

**Affiliations:** 1 The Nelson Mandela African Institution of Science and Technology, P.O.BOX 447 Arusha, Tanzania; 2 Ifakara Health Institute, Health Systems and Policy Review & Environmental Health and Ecological Sciences (IHI), Kiko Avenue, Mikocheni, P. O. Box 78373, Dar es Salaam, Tanzania; 3 Ifakara Health Institute, P.O. Box 78373 Dar es Salaam, Tanzania

**Keywords:** community health workers, maternal and neonatal health, challenges, Bagamoyo district, Mkuranga district

## Abstract

**Objectives:**

Community health workers (CHWs) play significant roles in areas where professional health workforce succumb to serious human resource deficiencies. This study explored challenges the CHWs face in promoting maternal and neonatal health in two districts of Tanzania.

**Design:**

A cross-sectional study design was conducted in Bagamoyo and Mkuranga districts from March to May 2019.

**Methods:**

Qualitative data were collected using in-depth interviews from 30 CHWs, thematic analysis was carried out by identifying major key themes emerging from the data.

**Results:**

The study found little community support resulted from jealousy of male household heads, mistrust, social-cultural believes and lack of community appreciation on the role of CHWs. Little support from local government resulted by political grievances among local leaders due to change in political administration. Other challenges were lack of basic knowledge in maternal and neonatal health, including breastfeeding. Irregular working schedules due to poor supervision, huge workload, old age and tiredness

**Conclusion:**

CHWs programs in Bagamoyo and Mkuranga districts were inactive and required innovative strategies to raise community and local government cooperation. Jealousy among male household's heads and CHWs need community members to participate in establishment of selection criteria. Socio-cultural beliefs call for community sensitization on how they affect maternal and neonatal health. Lack of community appreciation on the role of CHWs, irregular working schedule implied poor supervision and defined age limit would overcome challenges related to old age.

**Funding:**

The study received financial support from the African Development Bank (AfDB) through a master scholarship program at the Nelson Mandela African Institution of Science and Technology (grant number. SA_Z1_21001550338)

## Introduction

Shortage of human resource for health in maternal and child health has led to increasing Community Health Workers (CHWs) in middle and low-income countries.^1^ Shortage of health workers threatens the achievement of Sustainable Development Goal number 3 that encompasses good health and well-being of all people, including reduction of maternal and neonatal mortality by less than 70 per 100,000 births and 12 per 1,000 births, respectively in 2030.^2^ The reported global maternal mortality rate is 211 per 100,000 live births in 2017 and high in Sub-Saharan African countries with a rate of 542 deaths per 100,000 live births.^3^ In 2018, approximately 2.7 million neonates died Worldwide, with Sub-Saharan Africa reported the highest neonatal mortality rate of 28 deaths per 1,000 live births.^4^ Maternal mortality rate in Tanzania is 556 deaths per 100,000 live births, while neonatal mortality rate is 25 per 1,000 live births.^5^. Hence, maternal and newborn health is a challenge fueled by inadequate access to health services, mostly in rural areas.^6^

Seventy per cent of Tanzanians live in rural areas where health systems experience a shortage of health care providers, poor transport, long distance to health facilities and inadequate medical supplies.^7^

Tanzania opted to fill the gap by using CHWs to promote community-based health care services, including maternal health, since 1960s. Still, the program declined in 1990s due to poor performance and donor influence on adoption of disease-specific programs.^8^ In 2007-2017, Tanzania launched “*Mpango wa Maendeleo wa Afya ya Msingi*” (MMAM) to increase community-based health care where the importance of using CHWs surfaced for the second time.^9^ Health actors, including non-governmental organization, the President's Emergency Plan for AIDS Relief (PEPFAR) and African Medical and Research Foundation (AMREF), launched CHWs programs.^8^, ^10^ In addition, the National Health Policy of 2017 endorsed use of CHWs to promote maternal and neonatal health at the community level.^11^ Likewise, the Ministry of Health, through their Health Sector Strategic Plan of July 2015 to June 2020 have categorically stated that mobilization and training of CHWs is among six initiatives launched to realize a 20% reduction of maternal and neonatal mortality by 2017/18.^12^

Community health workers secured a trusted position to bridge between the formal health system and the community.^13^ Their main designated role is to promote utilization of services provided by the formal health system at household levels. In Bagamoyo and Mkuranga districts, between 2007-2017, CHWs were selected and trained for 3 to 24 days to provide services at household level on water, hygiene and sanitation, child survival and reproductive health interventions, as well as providing support in collection of information and report writing.^8^

Community members following criteria set by Ward development committee and program team from non-governmental organizations selected CHWs. Among the selected criteria is the ability to read and write, residing in respective village, willingness to work as a volunteer and acceptability of an individual by community members.^10^, ^14^ After selection and training, CHWs were deployed to provide services under programs that were run by nongovernmental organizations 10. However, after the phaseout of the program, CHWs were handed to local governments for sustainability^14^. No study has been done to assess their progress to date. Several studies have indicated their general role in child survival and reproductive health. However, it is unclear what kind of services they specifically provide in maternal and neonatal health sectors in Bagamoyo and Mkuranga districts in Tanzania.

Bagamoyo and Mkuranga districts have registered neonatal mortality rate of 53 per 1,000 live births and 52 per 1,000 live births, respectively. In both districts, neonatal mortalities contribute to over 67% of total under-five deaths.^15^ In 2007, Mkuranga district was reported to have high maternal mortality of 320/100,000 live births 16 and Bagamoyo district council reported maternal mortality rate of 204/100,000 live births in its strategic plan of 2016/2017-2020/2021.^17^ Despite the unavailability of current statistical data on maternal mortalities in Mkuranga district, studies have indicated high maternal deaths.^18^ To address maternal and neonatal deaths, Bagamoyo and Mkuranga districts have been using CHWs who were handed to local governments by nongovernmental organizations since 2007 to date^14^ as one of the national strategies.^12^

Studies in Mkuranga and Bagamoyo districts have shown significant involvement and contribution of CHWs in delivering maternal health interventions. Mobile health among CHWs was reported to increase awareness of danger signs among pregnant women in the Bagamoyo district.^19^ Unfortunately, despite their contribution, studies have continued to report high maternal and neonatal deaths in these districts, thus call for the need to evaluate other influencing factors that contribute to that rate. Literature has indicated lack of evidence on CHW experiences, including challenges in providing maternal and neonatal health care in these districts and Tanzania in general.^10^ A study in Mkuranga reported poor community recognition of CHWs roles and existence.^20^ However, the study did not explore CHWs views and experiences.

It is suggested communities to have chances to select and set mechanisms to support CHWs and future research to examine the community's contribution towards the functioning of CHWs.^21^ Similarly, a study in Uganda indicated compatibility of CHWs program with social-cultural beliefs enhanced positive acceptability by the community members. But it as well reported negative program acceptability in some areas due to undesirable behaviour and lack of trust expressed by CHWs.^22^

A study that indicated CHWs conflicts with some community members was reported in Brazil,^23^ although it did not examine the workload and time management of CHWs. Another study revealed the need to explore the blockage of integrating CHWs into health care systems due to lack of financial incentives, motivation and collaborative and unsupportive working environments.^24^ Shortage of financial incentives decreased performance and affected volunteerism of CHWs in most of African countries.^25^, ^26^ Studies in Uganda, Nigeria, and Tanzania have corroborated the findings on CHWs' prospective to promote effective health services to their areas by integration of the program into policy and provision of essential training and supplies.^24^, ^27^-^30^ Similarly, understanding of CHWs workload, community acceptability, the time they allocate to volunteer services, assigned roles and responsibilities are essential for better CHW management and performance^24^ but such information is lacking in studies done so far in Tanzania. Therefore, this study aimed to fill the knowledge gap by exploring several challenges and experiences of CHWs and how they promote maternal and newborn health interventions in Bagamoyo and Mkuranga districts in Tanzania based on CHWs' perspectives.

## Methods

### Study Design and Settings

A cross-sectional study design was deemed appropriate for this kind of study to get an inner picture of CHWs challenges during implementation of their assigned duties in two districts of Bagamoyo and Mkuranga in Pwani region in Eastern part of Tanzania. The districts are located between 60° and 80° South of the equator and between 370 – 40010′ East of the Greenwich Meridian line.^31^ Qualitative interview was conducted between March and May in 2019. The total population of the surveyed districts was 534,661 by the time of the research.^32^ The health services delivery is dominated with public health systems comprising of dispensaries, health centers and hospitals.

### Sampling

**Figure 1:**
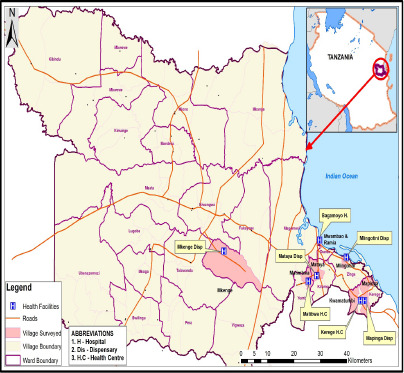
A map of Bagamoyo district showing selected health facilities and surrounding villages

CHWs were purposefully selected to include those who provide maternal and child health services. Purposeful sampling was used in participant's selection because, CHWs had experiences in promoting maternal and neonatal health services at the community level. CHWs were recruited based on their availability and activeness in provision of maternal and child health services. The shortage of health care providers in rural settings including Bagamoyo and Mkuranga districts necessitated to incorporate at least two trained CHWs to work in the Reproductive and Child Health section (RCH). They assist in day-to-day activities such as cleanliness, weighing and recording of children's weight, set up appointment for next visits in the maternal and newborn clinics. Therefore, participant's recruitment was done through public health facilities including hospitals, health centers and dispensaries because CHWs were easily available through facility in-charges than finding them in the community. The study sought phone numbers of active CHWs from health facility in-charges who supervised them and scheduled an interview at their homes. All public hospitals and health centers were included in sampling frame for identification of participants due to its small number. Dispensaries were randomly selected among many available in the study areas. Bagamoyo district consisted of 27 health facilities including both public and private, there is one district hospital, four health centers, one maternity center, one child specialized clinic and twenty dispensaries. Fifteen CHWs in Bagamoyo district were interviewed (see Table 1). Three CHWs were selected from the district hospital, six from two health centers and six from three dispensaries as summarized in Figure 1.

**Table 1:** Social-demographic characteristics of community health workers

Variable	Number of CHWs interviewed Bagamoyo district n(%)	Number of CHWs interviewed Mkuranga district n(%)	Total number of CHWs in both districts n(%)
**Sex**			
**Male**	6(40%)	11(73%)	17 (57%)
**Female**	9(60%)	4(27%)	13(43%)
**Age (years)**			
**20 – 50**	6(40%)	8(53%)	14(47%)
**51-70**	9(60%)	7(47%)	16 (53%)
**Marital status**			
**Unmarried**	6(40%)	1(7%)	7(23%)
**Married**	9(60%)	14(93%)	23(77%)
**Level of education**			
**Standard 7**	15(100%)	15(100%)	30(100%)

**Figure 2:**
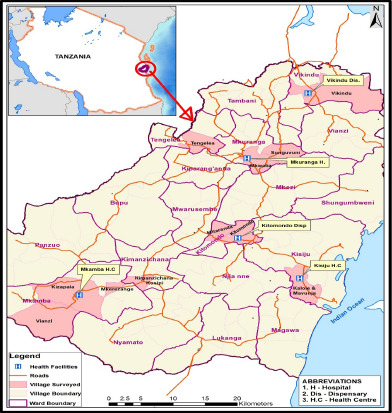
A map of Mkuranga district showing selected health facilities and villages surrounding

Fifty-four health facilities were included in sampling frame in Mkuranga districts. These facilities included both, public and private with one district hospital, six health centers and forty-seven dispensaries. Likewise, fifteen CHWs (see Table 1) were interviewed including, three from the locality of district hospital, six from health centers and six from dispensaries (Figure 2). Saturation point was attained by interviewing 30 CHWs as shown in Table 1.

### Ethical Approval

Ethical approval was granted by Ifakara Health Institute's Institutional Review Board with an identification number IHI/IRB/NO: 3-2019. Research permits to visit study areas were obtained from Bagamoyo and Mkuranga district councils before data collection. Informed consent was obtained from CHWs by signing a consent form to confirm their participation. However, clarification on study purpose and rights of participants was done prior to joining the study. CHWs were given right to terminate participation at any time without harm.

### Methods of data collection and information collected

Thirty in-depth interviews were conducted with CHWs in Bagamoyo and Mkuranga districts. The study collected information related to personal experience and challenges of CHWs when implementing interventions in maternal and newborn health. Preliminary information including place of residence, sex, education level, age and marital status were used to inform the social demographic characteristics. Variables used to capture CHWs challenges expressed in this paper were exploration of challenges they face when implementing their work including when delivering maternal and neonatal services at households. Exploring if they received refresher training after deployment and exploring some basic knowledge in maternal and newborn health such as breastfeeding. Other variables were exploration of support they get when implementing their work, exploring their responsibilities to understand specifically the package of maternal and neonatal health services they provide. Exploration on working schedules to understand the time dedicated for health service provision in household visits.

### Data Analysis

Audio-recordings were transcribed using Microsoft word and transcripts were translated from Swahili to English. Transcripts were rechecked for consistence with audiorecordings to ensure accurate capturing of information and correction of identified mistakes. Thematic analysis was done to identify key themes and sub-themes from data using framework method where all themes and codes where arranged in excel software. Four major themes were identified to guide the analysis process of the collected data. All authors had reached consensus on themes and sub-themes through discussion on significance of each theme in relation to the study aim and contribution to CHWs performance and general health system.

## Results

Results were organized into social-demographic characteristics and the reported challenges facing CHWs.

### Social-demographic characteristics

Table 1 shows the social-demographic characteristics of CHWs in Bagamoyo and Mkuranga districts. Findings revealed that majority of respondents were male aged 51-70 (53%). In both districts 23 (77%) were married and all 30 (100%) CHWs in this study were “standard seven” school leavers.

### Challenges facing CHWs in promoting maternal and neonatal health

Three major themes were deduced from responses of interviews conducted to CHWs these were lack of community support and appreciation of CHWs role; lack of local government support to CHWs; lack of basic knowledge on maternal and neonatal health. Other themes were irregular working schedules, heavy workload, old age and tiredness.

### Lack of community support and appreciation of CHWs role

The study realized CHWs challenges from their communities such as jealousy, mistrust and social cultural beliefs and lack of appreciation on CHWs roles. The following are explanations from CHWs that shows how the community posed challenges to CHWs work.

### Jealousy among CHWs and male household heads

Some male household members were hesitancy to CHWs services. A CHW respondent revealed that a certain male household-head was suspicious of entertaining mischievous behavior between his spouses with a male CHW.

*“Some men are jealous and reluctant to receive health education. Sometimes they abused us and say that are tired of our services. Sometimes, I felt disappointed to continue visiting households and decided to go back home (CHW, Mkuranga district)”*.

They refused them to visit their homestead for fear that male CHWs would indulge in extramarital affairs with their wives and/or daughters.

### Mistrust between CHWs and community members

The most dreaded issues were that of HIV-status' disclosure and home visits of CHWs. A response from one CHW of Bagamoyo district on challenge they face was that:

*“Pregnant women do not like to disclose their HIV status. They claimed that CHWs are visiting their homes to know if they are HIV positive so that they can disclose to other people. Sometimes, they say they do not have HIV (CHW Bagamoyo district)”*.

CHWs reported high level of community mistrust regardless of acceptability that was shown by community members during recruitment process. Most pregnant HIV positive women fear that CHWs would expose to public their HIV status. Underlying, the communities have little trust to CHWs practitioners. One CHW respondent reported that they were not welcomed to homes due to lack of identity cards and uniforms:

*“We do not have identity cards and new people from other place migrate into our village daily. Once I visited a household with no identity card and a uniform, I was not welcomed*.

*We used a lot of time to introduce ourselves but they refused to accept our services. (CHW, Bagamoyo district).”*

### Social cultural beliefs

On the side of female house members, especially expected mothers, the study found that most do not show cooperation with CHWs as they have ill feelings against purported witchcraft practices in their communities.

*“Sometimes pregnant women do fear disclosing their pregnancy status to avoid being witched by their enemies. Likewise, pregnant women who are almost due are moved to other places to conceal their whereabouts*.

*They believed that CHWs could be used to expose their status and risked being harmed” (CHW, Mkuranga district).”*

### Community appreciation of CHWs roles

The study has noted that CHWs role are not fully appreciated and adhered as often are excluded from exercising their line of duties.

*“I am not consulted when mothers and neonates faced challenges after delivery. Usually women deliver at health facility and go back home, if it happens the newborn face minor problems, like non-stop crying, I am able to give my advice. But, this would depend on being informed that there is a newborn somewhere in my village because my village is too big. Sometimes children are born without my knowledge and sometimes I am informed but fail to heed the call for lack of transport (CHW, Mkuranga district).”*

Failure to notify CHWs about newly born presence of neonates around their jurisprudence or quickly discharged delivery women from the health facilities hinders effective provision of CHWs neonatal services in the surveyed districts.

### Local government support

CHWs in Mkuranga reported to receive little support from village government because of political affiliation. Change in political administration tends to enforce changes of new CHWs. The situation has swayed the recruitment of CHWs to be partisan oriented. A claim from one CHW revealed the following complexity:

*“I felt to get support from local leaders because I was selected when our village was under the ruling part CCM (Chama Cha Mapinduzi). Now it is under CUF (Civil United Front). CUF wants to select new CHWs but the district medical officer rejected their proposal on the pretext of high cost of training. I would normally inform village chairperson about my plan to make homes visit but once I do so, the village chairperson passes around each household to persuade them not to cooperate with me. (CHW, Mkuranga district).”*

The partisan based CHWs recruitment in the surveyed areas indicates the extent of animosity between CHWs and local government leadership:

*“When we communicate our challenges to village government we are told they are related to “CCM party” we are not given allowance since CUF acquired village leadership in our village (CHW, Mkuranga district).”*

### Basic knowledge on maternal and neonatal care

When asked about given opportunities to update their skills, majority of CHWs reported that last time they have attended refresher training course was in 2016. We specifically measured the knowledge of breastfeeding to attest their knowledge of maternal and neonatal care. The response from inexperienced was:

*“Breast milk are not enough for the baby under six months, complementary food is required because babies do cry demanding for extra food from the first month and are born with hunger” (CHW, Mkuranga district).”*

This response goes against the WHO's standard^33^, ^34^ on infants and young child feeding practices. For the matured and experienced CHW, the response was:

*“We insist mothers to breastfeed babies without feeding them with anything for six months, but they do ask as to why their children are not given water. You can find a newborn given water within two weeks after delivery and this make some newborn experience stomach pains” (CHW, Bagamoyo district).”*

### Irregular working schedule and concentration on income generating activities

In responding to the questions if they find satisfied with their jobs and assistance given or whether they feel overwhelmed, the respondents pointed on lack of motivation to spend most of their time on income generating activities.

*“The time that I use to provide services as CHW does not exceed six hours and it is not every day, it depends on the availability of the task. As a CHW, I just volunteer so I cannot provide service every day, I have other family responsibilities (CHW, Bagamoyo district).”*

Additionally, although CHWs are working under the Ministry of Health and supervised by health facility incharges managed by the District Medical Officer. They are not paid any allowances or salaries. Visits to homesteads are done irregularly with no appointments or strict schedules

*“During visits, I can reach at the household, but I cannot find the owners of the household. I just make visits without knowing presence of family heads in my village, I cannot even make a call because I do not have their mobile numbers (CHW, Bagamoyo district).”*

### Old age and tiredness

Some CHWs who have become older cannot perform well due to physical inability. Most of these CHWs said, they had worked for long time and wanted to retire. Some CHWs reported to lose eyesight and they are not able to fill in working documents thus affect their general performance.

*“I face a challenge to fill in the documents because my eyes have lost vision as I am becoming older. Sometimes I get confused” (CHW, Bagamoyo district)*.

*“Honestly, I am too old, I had requested the authority to allow me to retire but they rejected” (CHW, Mkuranga district)*.

### Unbearable workload

Reports of heavy workload cut across all districts. For example a village in Mkuranga district has 670 households and a population of about 2847 people^32^ but is served by only one CHW. In Bagamoyo district, one village with 289 households and a population of about 1129 people is served by one CHW. All these factors contributed to severe burnout and hence demonstrated the presence of some demotivated CHWs in both districts.

*The work is very tough because I am working alone, my colleague has been selected recently but she is not aware of many things related to maternal and newborn health, she cannot even answer your questions” (CHW, Mkuranga district).”*

As for total burnout, the following response by CHW at Mkuranga district was found to sum the matter.

*“First of all the challenge is that, my village is very large consisting of five sub-villages. I walk by foot alone to provide services to the community (CHW, Mkuranga district).”*

## Discussion

The study noted major challenges that CHWs succumb at Mkuranga and Bagamoyo in Pwani region in Eastern coast of Tanzania. The socio-demographic profile of 30 CHWs respondents indicates that majority are male and have only basic primary level education. About 53% of CHWs workforce are at the age range of 51-70 that can affect their productivity and learning of new skills. The younger generation though still a good number (47%) ranging from 20-50 years but have no proper training in maternal and neonatal health knowledge.

The current study has shown that there is minimal liaison between CHWs and heads of families at village levels. Mistrust ensues them for fear of promiscuity and other mischievous behavior such as extra-marital encounters. Similarly, the result of mistrust between CHWs and household male and female heads is also reported in Uganda and South Africa.^22^, ^35^ To overcome such suspicious relation and build trust between CHWs and people they serve, criteria for selection should be co-designed and co-implemented by the communities, health practitioners and Ward development committee as suggested in the contextual and empirical study.^14^ To avail fear of breaching of confidentiality for pregnant women, who are HIV positive, scrutiny of ethical sounding candidates for CHW post must be emphasized. Similar results on fear of breaching of confidentiality was reported in South Africa and China.^35^ ^36^

When it comes to lack of identity cards and uniforms, similar cases were reported elsewhere.^22^, ^35^ Mainstreaming of CHWs is not an option as they are under the Ministry of Health and backed by relevant policy to incorporate CHWs in the health delivery chain. It should not only end in formal recognition by government organs in terms of identity cards and uniforms but should go further to consider remuneration (allowances or salaries) to boost their low morale. Identity cards and uniform must be used by CHWs during household visits to build trust and increase their acceptance in the community.^13^, ^27^

To avoid political grievances or partisan prejudice as reported in this study, it is recommended to treat affairs of CHWs as purely non-partisan and should be at the hand of local governance.^10^, ^14^ The political grievances expressed in this study indicates weakness in interpretation of integration of CHW strategy in health systems.^37^

Furthermore, it is suggested that CHWs program sustainability should not end up handing over program to Local Government without support in training, allowances and supervision of CHWs.^38^ The lack of appropriate trainings in maternal health services on majority of CHWs in Bagamoyo and Mkuranga districts is an indication of the bigger picture characterizing the whole country and as well in other low-and-middle income countries about the value of CHWs and their contributing factor in the primary health system. It is serious when discovering that CHWs are delivering wrong message to mothers on breastfeeding and complimentary feeding protocol. WHO recommends exclusive breastfeeding below 6 months and complementary feeding afterward to maximize early childhood growth and development. Exclusive breastfeeding is the provision of only breast milk to infant without given other liquid, solid even water, with the exception of oral rehydration solution, syrups of vitamins, minerals or medicines for optimal growth, development and health. Whereas complementary feeding is the introduction of nutritionally appropriate and safe foods to infants aged 6 months and above to supplement breastfeeding.^33^, ^34^ Thus, any introduction of complementary foods earlier than the recommended 6 months of age, which is a common practice in Tanzania, is wrong approach and indicates lack of maternal and neonatal appropriate feeding knowledge on the part of CHWs. This is contrary to what has been shown in the literature about the potential role of CHWs at early postnatal period, including assessment of maternal and newborn danger signs, promoting hygiene practices and promotion of awareness and practices on exclusive breastfeeding on infants and young child between the age of 0-6 months.^39^, ^40^

The study also noted the presence of irregular working schedules caused by poor supervision. Majority of CHWs were mainly working on income-generating activities such as designing clothes as reported by the current study. This was consistent with other studies reported elsewhere.^41^ But findings differ to others reported in Ethiopia, where Health extension workers used most of their time in delivering services to households.^42^

### Limitation of the Study

Findings might have been affected by information bias including CHWs ability to recall information regarding questions that were asked and selection bias. Since the study recruited only active CHWs to gather their experiences and challenges in providing maternal and neonatal health. The study was unable to recruit inactive CHWs because their phone numbers were not available, others had stopped providing services and some were newly selected in such a way that could not answer the study questions thus call for future research to investigate reasons to why other CHWs are inactive and no longer providing services. However, sample size is not a limitation to this study since saturation was attained by interviewing thirty CHWs.

## Conclusion

To promote maternal and neonatal health, CHWs require both community and government support. Despite the existing challenges in implementing CHWs programs within the health systems, the Central Government commitment through the Ministry of Health particularly the District Medical Office and health facility in-charges, Local Governments through local leaders, identification of roles and responsibilities particularly in maternal and neonatal health, motivation and supervision is of essence. Likewise, it is important to avoid political grievances as reported in this study. It is recommended to have by-laws governing CHWs insisting on non-partisan local government ethos. Refresher trainings to improve CHWs knowledge especially in neonatal health is important. Likewise, community cooperation may help to minimize challenges such as mistrust and enhance smooth operation and performance of CHWs at the village or community level.
